# A Tree Peony Trihelix Transcription Factor PrASIL1 Represses Seed Oil Accumulation

**DOI:** 10.3389/fpls.2021.796181

**Published:** 2021-12-10

**Authors:** Weizong Yang, Jiayuan Hu, Jyoti R. Behera, Aruna Kilaru, Yanping Yuan, Yuhui Zhai, Yanfeng Xu, Lihang Xie, Yanlong Zhang, Qingyu Zhang, Lixin Niu

**Affiliations:** ^1^College of Landscape Architecture and Arts, Northwest A&F University, Yangling, China; ^2^Oil Peony Engineering Technology Research Center of National Forestry Administration, Yangling, China; ^3^Department of Biological Sciences, East Tennessee State University, Johnson City, TN, United States; ^4^Academy of Medical Sciences, Zhengzhou University, Zhengzhou, China

**Keywords:** PrASIL1, transcription factor, seed oil, tree peony, fatty acid biosynthesis

## Abstract

In many higher plants, seed oil accumulation is governed by complex multilevel regulatory networks including transcriptional regulation, which primarily affects fatty acid biosynthesis. Tree peony (*Paeonia rockii*), a perennial deciduous shrub endemic to China is notable for its seed oil that is abundant in unsaturated fatty acids. We discovered that a tree peony trihelix transcription factor, PrASIL1, localized in the nucleus, is expressed predominantly in developing seeds during maturation. Ectopic overexpression of *PrASIL1* in *Nicotiana benthamiana* leaf tissue and *Arabidopsis thaliana* seeds significantly reduced total fatty acids and altered the fatty acid composition. These changes were in turn associated with the decreased expression of multitudinous genes involved in plastidial fatty acid synthesis and oil accumulation. Thus, we inferred that PrASIL1 is a critical transcription factor that represses oil accumulation by down-regulating numerous key genes during seed oil biosynthesis. In contrary, up-regulation of oil biosynthesis genes and a significant increase in total lipids and several major fatty acids were observed in *PrASIL1-*silenced tree peony leaves. Together, these results provide insights into the role of trihelix transcription factor PrASIL1 in controlling seed oil accumulation. *PrASIL1* can be targeted potentially for oil enhancement in tree peony and other crops through gene manipulation.

## Introduction

In many higher plants, the seed storage reserves include oils as triacylglycerols (TAGs), carbohydrates, and storage proteins. Seed oils not only provide an essential energy source to support post-germinative growth and subsequent seedling development but also serve as the main source of nourishment for humans and farm animal (Li et al., 2006; Graham, 2008). Currently, oils are utilized by a broad variety of industries as well, such as raw materials for the manufacture of pharmaceuticals and biofuels (Durrett et al., 2008; Egert et al., 2014). As such, knowing the regulatory function of critical genes in seed oil accumulation is of significant basic and strategic benefits.

The accumulation of TAG is a complex biochemical process involving two subcellular organelles (Bates et al., 2013). The synthesis of fatty acids (FA) is initiated in the plastid in a multistep process with the involvement of many key enzymes. Plastidial pyruvate dehydrogenase complex (PDHC) first catalyzes pyruvic acid transformation to acetyl-CoA, and then 3-ketoacyl-ACP synthases (KAS catalyzes the condensation reactions (Johnston et al., 1997; Pidkowich et al., 2007). Subsequently, FAs are transferred to the endoplasmic reticulum as acyl-coenzyme A (FA-CoA), and some undergo desaturation catalyzed by oleate desaturase (FAD2) and linoleate desaturase (FAD3) (Browse et al., 1993; Okuley et al., 1994). Finally, FA-CoAs are esterified with glycerol-3-phosphate (G3P) to complete the assembly of TAG. This process is accomplished by successive catalyzation by glycerol 3-phosphate acyltransferase (GPAT), lysophosphatidic acid acyltransferase (LPAAT), phosphatidic acid phosphatase (PAP), and diacylglycerol acyltransferase (DGAT). Additionally, phospholipid: diacylglycerol acyltransferase (PDAT) can also catalyze an acyl group from the sn-2 site of phosphatidylcholine (PC) to DAG to synthesize TAG (Baud and Lepiniec, 2010).

Transcriptional regulation is an important means to control gene expression for seed oil accumulation. Some of the transcription factors (TFs), referred to as master regulators, besides regulating the expression profile of biosynthetic genes, also the action of other TFs (Li et al., 2017). The master regulators such as WRINKLED1 (WRI1), FUSCA3 (FUS3), LEAFY COTYLEDON1 (LEC1), LEC2, and ABSCISIC ACID INSENSITIVE3 (ABI3) govern the seed maturity as well as oil synthesis process (Weselake et al., 2009; Baud and Lepiniec, 2010; Troncoso Ponce et al., 2011). Primarily, WRI1 alters the oil content by upregulating glycolytic and plastidial FA biosynthesis genes (Cernac and Benning, 2004; Baud et al., 2009; Kong and Ma, 2018; Kong et al., 2020b). Deletion of *WRI1* led to an 80% decrease in Arabidopsis seed lipid level (Focks and Benning, 1998), and conversely, overexpression of *WRI1* significantly increased the oil level (Maeo et al., 2009; Sanjaya et al., 2011; Adhikari et al., 2016). ABI3, FUS3, and LEC2 (AFL) as members of the B3 domain family, which have a B3 DNA-binding domain (Luerssen et al., 1998; Stone et al., 2001; Santos-Mendoza et al., 2008). These master regulators bind to the RY elements in the promoter region of the target genes involved in FA biosynthesis, and TAG storage protein synthesis to promote lipid yield (Reinders et al., 2002; Baud et al., 2016; Zhang et al., 2016). Activation of *LEC1* also upregulates a suite of genes contributed to glycolysis, and FA biosynthesis, elongation and desaturation (Mu et al., 2008). Both *LEC1* and *LEC2* are positive modulators of *WRI1*, *FUS3*, and *ABI*3 upstream (Kagaya et al., 2005; Weselake et al., 2009). Furthermore, there is a positive feedback loop among *ABI3* and *FUS3* that ensures continuous expression of themselves and each other (To et al., 2006). Moreover, some positive regulators of FA synthesis and oil accumulation, including MYB92, MYB96, and tandem CCCH zinc finger protein, GmZF351 and GmZF392 were uncovered (Lee et al., 2018; To et al., 2020; Lu et al., 2021). In addition to the positive regulators, there are also several TFs implicated in the inhibition of seed lipid synthesis. A group of B3 domain family TFs including VIVIPAROUS1/ABI3-LIKE1 (VAL1), VAL2, and VAL3 repress oil accumulation by downregulating the AFL genes and their target genes of the network (Tsukagoshi et al., 2007). The CHD3 chromatin remodeling factor PICKLE (PKL) also can downregulate the members of the AFL network, thereby contributing to the shutdown of the seed maturation program (Ogas et al., 1999; Zhang and Ogas, 2009). The Arabidopsis 6b interacting protein 1-like 1 (ASIL1) inhibits the transcription of embryonic maturation genes in Arabidopsis seedlings and acts downstream of the miRNA to restrict seed maturation (Gao et al., 2009; Willmann et al., 2011). MYB89 and MYB76 were also investigated as two inhibitors of seed oil accumulation. Recently, a novel FA synthesis repressor, TEOSINTE BRANCHED1/CYCLOIDEA/PROLIFERATING CELL FACTOR4, was investigated to inhibit the expression of fatty acid biosynthetic genes by interacting with WRI1. These finding is of great importance for advancing the study of transcriptional regulatory networks of seed oil accumulation (Duan et al., 2017; Li et al., 2017; Kong et al., 2020a).

Tree peony (*Paeonia* section *Moutan* DC.), a perennial deciduous shrub endemic to China, has been cultivated for ornamental and medicinal usage for over 2000 years (Zhou et al., 2014). Recently, tree peony seed oil gained prominence as a rich resource for unsaturated fatty acids (UFAs > 90%) such as linoleic acid (LA, ∼25%) and α-linolenic acid (ALA, ∼45%), which are essential to the human body (Li et al., 2015; Zhang et al., 2018). Thus, tree peony became an emerging woody oil crop in China and its seed oil has been authorized for human consumption since 2011 (Zhao et al., 2020). Consequently, tree peony cultivation has been promoted in China and attracted extensive attention as a novel oil crop.

Recent studies unveiled a host of genes engaged in seed oil biosynthesis and accumulation in tree peony (Yin et al., 2018; Zhang et al., 2019; Zhao et al., 2020). However, the role of TFs regulating oil accumulation in tree peony seeds is poorly understood. Previous transcriptome data revealed that a trihelix TF, *PrASIL1* is highly expressed in seeds during early seed development and maturation in tree peony. Although implicated in the temporal regulation of seed filling and maturation in Arabidopsis, its role in seed oil biosynthesis is not well-studied. In this study, we show that PrASIL1 acts as a negative regulator and its reduced expression is associated with upregulation of oil biosynthesis genes and oil accumulation in seeds until maturation in tree peony.

## Materials and Methonds

### Plant Materials and Growth Conditions

*Paeonia rockii* was grown at the wild tree peony germplasm repository of Northwest Agronomy and Forestry University, Shaanxi Province, China (34°16′ N, 108°4′ E). The seeds at 20, 40, 60, 80, and 100 days after pollination (DAP) and other organs, such as roots, stems, leaves, calyxes, petals, stamens, and pistils of *P. rockii* were collected into liquid nitrogen and then stored at −80°C. Additionally, 2-year-old seedlings at 4 weeks post-germination were selected for virus-induced gene silencing (VIGS). Both plants, *Nicotiana benthamiana* for transient expression studies and *Arabidopsis thaliana* (Columbia-0; wild type) for ectopic overexpression experiment were grown in the same climate chamber with 16h light/8h dark and 65% relative humidity at 22°C.

### Gene Cloning and Plasmid Construction

RNA was extracted from seeds or other organs using the RNA Prep Pure Plant kit (TIANGEN) and reverse transcribed using PrimeScript™ RT reagent Kit (TaKaRa). Specific forward and reverse primers (*PrASIL1-*F/*PrASIL1-*R) were used to amplify coding region of *PrASIL1*. The PCR products were purified by DNA Gel Extraction Kit (Sangon) and cloned into pMD19-T vector. The construct *35S:PrASIL1-GFP* for subcellular localization assay was generated by inserting the CDS of *PrASIL1* without the stop codon into pCAMBIA2300-GFP vector. The plasmids 2 × *35S*:*PrASIL1* used for transient expression assay in *N. benthamiana* and *35S*:*PrASIL1* used for stable overexpression experiment in *Arabidopsis thaliana* were constructed by inserting the CDS of *PrASIL1* into pB110 and pCAMBIA2300, respectively. To obtain the TRV2-*PrASIL1* construct for VIGS assay, the silencing fragment was amplified and connected to TRV2. The primers used for gene cloning and plasmid construction in this study which were designed by Oligo 6.0 software are listed in Supplementary Table 1.

### Phylogenetic Analysis and Conserved Motif Analysis

Previously, thirty trihelix genes of *A. thaliana* were reviewed in detail (Kaplan-Levy et al., 2012). Of these, protein sequences of nineteen representative trihelix genes of *A. thaliana* and *PrASIL1* were selected for multiple sequence alignment using Clustalx2.11.^1^ The sequences of *Arabidopsis* trihelix proteins were obtained from the TAIR database.^2^ A phylogenetic tree was generated in MEGA7.0 using the neighbor-joining (NJ) method with Poisson correction and 1000 replicates for bootstrap analysis. Furthermore, the MEME tool^3^ (Bailey et al., 2015) was used to identify and compare conserved motifs among PrASIL1 *P. rockii* and trihelix proteins of *A. thaliana*. The analysis parameters were set to the maximum number of motifs as 10 and the motif width of 6–50 aa.

### Subcellular Localization

*35S:PrASIL1-GFP*, as described previously was used for localization studies. *35S:NbWRKY8-mCherry* was used as a positive nucleus marker (Ishihama et al., 2011). They were transformed into onion epidermal cells by particle bombardment, in which gold powder embedding was performed according to the BIO-RAD Biolistic PDS-1000/He system (BIO-RADCA, United States) (Huang and Liu, 2006). Onion epidermis (approximately 1.5 cm × 1.5 cm) was placed on MS solid medium and incubated at 22°C for 24 hours. Subsequently, onion epidermal cells were bombarded using a particle bombardment with a rupture disk pressure of 1.1 kPa and distanced at 5cm. After incubation for at least 15 h at room temperature in the dark, the onion epidermal cells were then observed under a confocal laser scanning microscope (UltraVIEW VoX, PerkinElmerafter). GFP was excited by a 488 nm laser, the emission was obtained in 560–620 nm. The mCherry was excited by a 552 nm laser and acquires emission at 600–650 nm. The dimensions of the visual field were 237.42 × 237.42 μm^2^.

### Transient Overexpression in *Nicotiana benthamiana* Leaves

The *Agrobacterium tumefaciens* strain LBA4404 was transformed with pB110 binary vector carrying *35S:PrASIL1* using the freeze-thaw method (Behera et al., 2021), and positive colonies were selected on solidYEP medium with kanamycin (50 μg/mL) and rifampicin (50 μg/mL). The positive colonies were then subcultured in YEP liquid medium with the same antibiotics and culture d at 28°C with constant shaking overnight. The bacterial cells were then harvested by centrifugation at 400xg for 10 min and suspended in the infiltration buffer (300 μM acetosyringone, 10 mM MES, 10 mM MgCl_2_, pH 5.7) to a final OD_600_ of 0.6. The suspension was maintained in the dark for 3 h at 28°C before infiltration. To enhance the *PrASIL1* expression in the leaf tissue of *N. benthamiana*, suspension of *Agrobacterium* with P19 silencing inhibitor and suspension of Agrobacterium containing 35S:*PrASIL1* were mixed in a ratio of 1:1 and then infiltrated into 4-week-old tobacco leaves using a needleless syringe. Untreated leaves and leaves injected with *Agrobacterium* containing P19 served as the control group.

### Visualization of Lipid Droplets

To visualize lipid droplets (LDs) in *N. benthamiana* leaf tissues, LDs were stained with 2 μg/mL Nile Red (YUANYE) in 0.01 mM phosphate buffer saline (pH 7.2). Confocal images were obtained utilizing a laser scanning confocal microscope (SP8; Leica). Nile Red was excited by a 488 nm laser, the emission was obtained in 560–620 nm. The dimensions of each visual field were 232.50 × 232.50 μm^2^. Number of LDs counted per image area using ImageJ software. Three biological replicates were performed to visualize LDs in *N. benthamiana* leaf tissues.

### Stable Transformation of *Arabidopsis thaliana*

The *P. rockii 35S:PrASIL1* overexpression plasmid was constructed by pCAMBIA2300 and transformed into *A. tumefaciens* strain GV3101. Subsequently, GV3101 expressing *35S:PrASIL1* was transformed into wild-type Arabidopsis plants using the floral dip method (Clough and Bent, 1998). The T0 seeds were germinated on 1/2 MS media with kanamycin (500 μg/mL) to select transformed lines. The positive lines were confirmed by amplifying the full length of *PrASIL1* using reverse transcription-PCR (RT-PCR) and their zygosity was determined if the independent lines of T1 generation showed 3:1 segregation on selection medium. These transgenic lines were grown up to T3 generation to obtain homozygous lines.

Mature *Arabidopsis* seeds were collected from the main inflorescence, especially from siliques that grow at the basal position. The seeds from several lines were randomly chosen and photographed utilizing a Nikon Eclipse 50i upright microscope.

### Virus-Induced Gene Silencing of *PrASIL1*

The TRV vectors TRV1 and TRV2 have been described previously (Liu et al., 2002). The *TRV2*:*GFP* and *TRV2*:*PrASIL1* (containing a 290 bp fragment of *PrASIL1*) constructs were transformed into GV3101 for VIGS assay. The transformed GV3101 were grown in YEP medium with appropriate antibiotics at 28°C with constant shaking overnight. The bacterial cells were harvested by centrifugation at 4,000 × g and resuspended in infiltration buffer as described previously (Xie et al., 2019), and kept in darkness for 4–6 h at 28°C. The *A. tumefaciens* infiltration buffer containing TRV1 and TRV2 vectors were mixed in a ratio of 1:1 to a final OD_600_ of 0.8 before infiltration into leaves of *P. rockii* seedlings. The leaves of WT and infiltrated with TRV2:*GFP* were used as controls. Six days post infiltration (DAI), the leaves were used for GFP expression analysis. At 14 DAI, the leaf samples from ten individual plants were collected for the determination of oil content.

### Fatty Acids Quantification

To quantify the FA content, 8 mg of Arabidopsis seeds or 50 mg of dried leaves of *N. benthamiana* and tree peony were used for each biological replicate. The extraction and analysis of FAs in leaf tissues (Li et al., 2012; Ji et al., 2018), and seeds were carried out as previously described (Chen et al., 2012). Briefly, total FAs were converted to FA methyl esters in a methanol solution containing 1 M HCl for 2 h at 80°C. FAs from leaves or seeds were subsequently measured utilizing a gas chromatograph (8890; Agilent). A capillary column (HP-INNOWax; 60 m × 0.25 mm I.D., 0.25 μm) with nitrogen as carrier gas (flow rate of 1.0 mL min^–1^) was used. The initiating temperature was 170°C and kept for 5 min, then raised to 210°C at a rate of 2°C min^–1^. The peaks of each FA species were identified corresponding to the FA methyl ester analytical standard (catalog number: CDAA-252795; ANPEL). Concentrations of FA species were normalized against the internal control heptadecanoic acid (ANPEL). Experiments were performed with three biological replicates.

### Expression Analysis

The expression of the transformed cDNA in transgenic plants was confirmed at the transcription level using reverse transcription-PCR (RT-PCR). Quantitative RT-PCR (qRT-PCR) was carried out for three biological replicates using SYBR^®^ Premix Ex Taq™ kit (TaKaRa). Three independent biological replicas are used for expression analysis. The 18S-26S internal transcribed spacer (*18S-26S ITS*) gene of *P. rockii*, *NbL23* of *N. benthamiana*, and *Atactin7* of Arabidopsis were used as an internal control to normalize the gene expression. The 2^–△△*CT*^ values were adopted to represent the relative expression levels (Livak and Schmittgen, 2001). The primers used for RT-PCR and qRT-PCR are listed in Supplementary Table 2.

## Results

### *PrASIL1* Expression in Tree Peony Seeds Is Reciprocal to Oil Accumulation

Previously, we performed RNA-seq analysis of tree peony seeds at three developmental stages and found that the transcript levels of genes related to plastid fatty acid synthesis, desaturation, and triacylglycerol assembly were high (Zhang et al., 2019). Subsequently, we also identified the function of *PrFAD2* and *PrFAD3* genes in ALA synthesis (Zhang et al., 2019).

**FIGURE 1:**
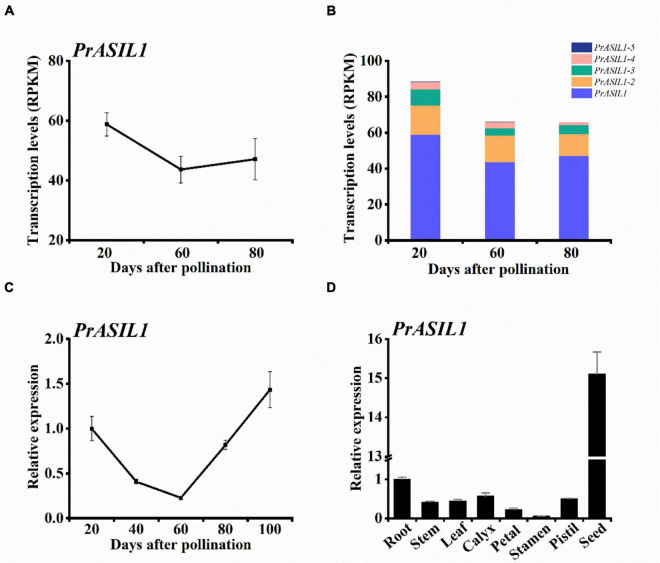
*PrASIL1* is highly expressed in developing seeds of *P. rockii*. **(A)** *PrASIL1* transcript levels in developing seeds. **(B)** Comparison of transcript levels of all *PrASIL1* paralogs in developing seeds. **(C)** Expression pattern of *PrASIL1* in developing seeds of *P. rockii* and **(D)** *PrASIL1* expression in various tissues of *P. rockii*. Values are mean ± SD (*n* = 3).

Transcriptomics also revealed that a trihelix TF PrASIL1 of tree peony was highly expressed in the early and late stages of seed development suggesting its association with seed-related traits (Figure 1A). Transcripts for additional paralogs of *PrASIL1*, named *PrASIL1-2*, *PrASIL1-3*, and *PrASIL1-4* with roughly similar expression profiles as *PrASIL1* were also identified (Supplementary Figure 1). Although, the expression levels for all *PrASIL1* paralogs were relatively high at 20 DAP, they decreased progressively with seed development (60 DAP). Among the paralogs, more than 50% of the transcripts expressed were contributed by *PrASIL1* (Figure 1B), suggesting a limited role for the remaining in seed development and oil accumulation. Therefore, we primarily selected *PrASIL1* as the target to further investigate its function in tree peony seed oil accumulation.

We further examined the expression pattern of *PrASIL1* in various developmental stages of tree peony seeds by utilizing qRT-PCR. The expression of *PrASIL1* at the 20 DAP and 100 DAP was higher that levels at 60 DAP (Figure 1C). This dynamic expression pattern of *PrASIL1* is reciprocally associated with the rate of fatty acid synthesis, which is at its highest during early to mid-developmental phase of the seed and plateaus by 100 DAP (Zhang et al., 2018). The transition of early to mid-maturation phase is marked by an increase in the storage lipid synthesis and thus a low expression of *PrASIL1* is desired. In contrast, the transition from mid to late seed maturation phase is accompanied with a metabolic switch in the seed tissue to increase the energy molecules such as sucrose biosynthesis to prepare the seed for germination. Thus, it is likely that PrASIL1 plays a part in the early and mature stages of seed development that is likely associated with initiation and termination of seed oil synthesis (Willmann et al., 2011).

We also examined the expression of *PrASIL1* in different organs including seeds at 100 DAP. Interestingly, a low-level expression of *PrASIL1* was noted in all the organs examined and relatively the expression was most abundant in matured seeds (Figure 1D). We predict that since the organs other than seed do not accumulate oil, PrASIL is not expected to play any regulatory role and thus its expression is likely maintained at a low level. It is also possible that other trans-regulatory factors might be suppressing the expression of ASIL1, including the positive regulators of lipid biosynthesis, because of the low oil content in these organs. In contrast, *PrASIL1* expression is dynamic in seeds as well as varying with development and oil biosynthesis.

### Molecular Characterization of PrASIL1

**FIGURE 2:**
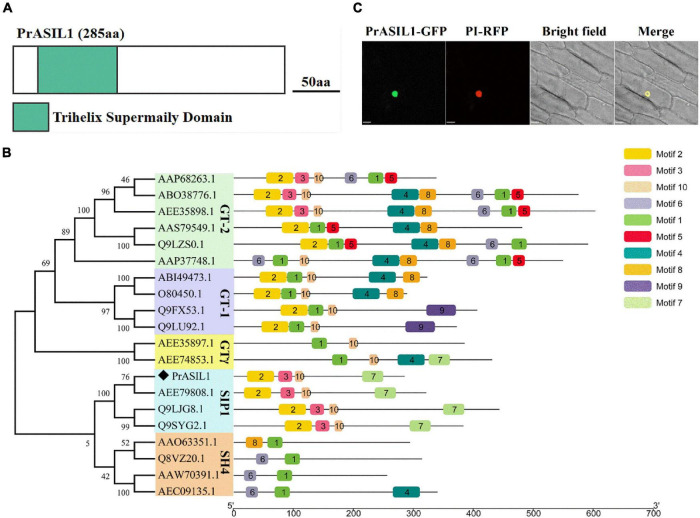
Molecular characterization of PrASIL1. **(A)** Schematic representation of PrASIL1 structure. aa, Amino acids. **(B)** Phylogenetic tree and conserved motifs of the PrASIL1. **(C)** Subcellular localization of the PrASIL1 protein fused with GFP (*35S:PrASIL1-GFP*) in onion epidermal cells. Bars = 33 μm.

The open reading frame of *PrASIL1* is 858bp and encodes for 285aa protein with one trihelix conserved domain and belongs to the trihelix family (Supplementary Figure 2 and Figure 2A). The trihelix family is divided into five sub-families based on the differences in their conserved domains (Kaplan-Levy et al., 2012). Phylogenetic analysis with select Arabidopsis trihelix TFs showed that PrASIL1 clustered into SIP1 subfamily. Comparison of the PrASIL1 with SIP1 TFs indicated four conserved motifs between them. Among them, only one conserved motif is located toward the C-terminal region, but the other three are located toward their N-terminal region. Notably, the conserved motifs of the trihelix TFs are mostly located at the N-terminal, suggesting it as the most conserved region (Figure 2B and Supplementary Figure 3).

To determine the subcellular localization of PrASIL1, a GFP fusion vector 35S:*PrASIL1-GFP* was constructed and transformed into onion epidermal cells. The results illustrate that PrASIL1-GFP co-localized with *35S:NbWRKY8-mCherry*, a nuclear transcription factor, indicating that PrASIL1 is nuclear localized (Figure 2C) and likely to function as a TF.

### Transient Overexpression of *PrASIL1* Reduces Oil Content and Changes Fatty Acids Profile in *Nicotiana benthamiana* Leaf Tissue

**FIGURE 3:**
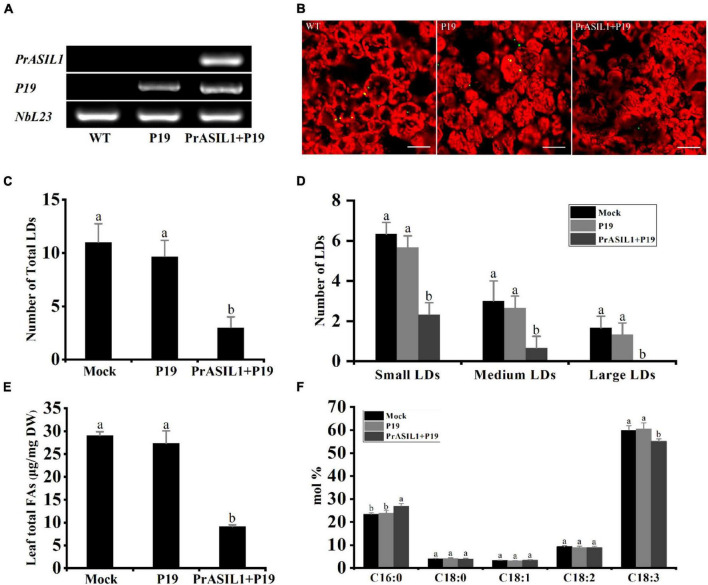
PrASIL1 reduces oil content and changes FA profiles in transformed *N. benthamiana* leaf tissue. **(A)** RT-PCR analysis of *PrASIL1* expressed in tobacco leaf tissue. **(B)** Representative confocal images of LDs in *N. benthamiana* leaf tissue. Green color shows LDs and red color shows chloroplast. Images are shown a 232.50 × 232.50 μm^2^ field of the leaf tissue. Bars = 40 μm. **(C)** Number of total LDs per image area in *PrASIL1-*transformed, mock, and P19-transformed *N. benthamiana* leaf tissue. **(D)** Number of LDs in different size categories per image area. Small LDs: Nile Red-stained lipid area < 3 μm^2^, Medium LDs: 3–6 μm^2^, Large LDs: 6–10 μm^2^. **(E)** Comparison of total FA content (μg/mg) among *PrASIL1-*transformed, mock, and P19-transformed *N. benthamiana* leaf tissue. **(F)** Fatty acid composition of *PrASIL1-*transformed, mock, and P19-transformed *N. benthamiana* leaf tissue. DW, Dry weight. Values are mean ± SD (*n* = 3). Different letters indicate significant difference at *P* < 0.05, as confirmed by one-way ANOVA with Tukey’s post-test.

The Agrobacterium-mediated transient expression is an advantageous system to study gene expression due to time-efficiency compared to stable transformation systems (Wroblewski et al., 2005). Additionally, several studies have successfully demonstrated that *N. benthamiana* leaf tissue can be used for transient expression of genes involved in FA synthesis and TAG accumulation to affect their metabolism (Grimberg et al., 2015; Ma et al., 2015, 2016; An et al., 2017; Snell et al., 2019; Behera et al., 2021). Here, we transiently expressed PrASIL1 under the control of dual CaMV 35S promoters in *N. benthamiana* leaves. The coding sequence for viral protein P19, an inhibitor of ectopic gene silencing, was also co-transformed. The expression of the *PrASIL1* was confirmed by RT-PCR, 6 days after leaf infiltration (Figure 3A). The overexpression of *PrASIL1* significantly decreased the number of lipid droplets (LDs), relative to the mock- and *P19*-transformed control leaves (Figures 3B,C). More specifically, overexpression of *PrASIL1* in leaves caused a considerable decrease in the numbers of small-, medium-, and large-sized LDs, compared to P19 control, by 58, 75, and 100%, respectively (Figure 3D).

Consistent with the number of LDs, the total FA content was also remarkably lower in the leaves transiently overexpressing *PrASIL1* than in the mock- and *P19*-transformed controls, suggesting a negative correlation between *PrASIL1* expression and oil biosynthesis (Figure 3E). Previous studies have shown that the change of oil content in tobacco leaf tissues was accompanied by an alteration of the FA composition (Vanhercke et al., 2014; Cai et al., 2015). Here, the expression of PrASIL1 resulted in the change of FA composition (Figure 3F). Specifically, the FA composition of C18:3 in *PrASIL1*-overexpressing lines was decreased to ∼89%, while the C16:0 was significantly enhanced, relative to the *P19*-transformed controls (Figure 3F). This reduction might be due to the inhibition of genes involved in FA biosynthesis and TAG deposition by overexpression of *PrASIL1*.

### Transient Overexpression of *PrASIL1* in Leaf Tissue of *Nicotiana benthamiana* Downregulates the Genes Involved in Fatty Acids and Triacylglycerol Biosynthesis

**FIGURE 4:**
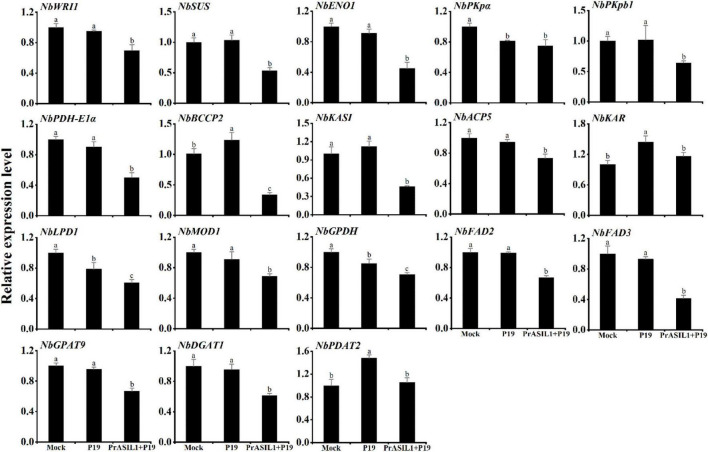
Comparison of the expression levels of genes involved in FA biosynthesis to TAG accumulation among *PrASIL1-*transformed, mock, and P19-transformed *N. benthamiana* leaf tissue. Results were normalized to the expression of *NbL23* and calibrated to the levels of mock tobacco leaves. Values are means ± SD (*n* = 3). Different letters indicate significant difference at *P* < 0.05, as confirmed by one-way ANOVA with Tukey’s post-test.

To investigate how PrASIL1 resulted in reduced oil accumulation, qRT-PCR was performed to quantitatively analyze the expression levels of several genes related to FA and TAG biosynthesis in the *N. benthamiana* leaves. Previous studies showed that the expression of *AtLEC2* in *N. benthamiana* leaves increased the transcription level of *NbWRI1*, a positive master regulator of FA biosynthesis (Nookaraju et al., 2014). Here, overexpression of *PrASIL1* decreased the expression level of *NbWRI1* (Figure 4). We further analyzed the expression levels of genes regulated by WRI1, including *SUCROSE SYNTHASE* (*SUS*), *PHOSPHOENOLPYRUVATE ENOLASE 1* (*ENO1*), *PYRUVATE KINASE ALPHA SUBUNIT* (*PKp*α), *PLASTIDIAL PYRUVATE KINASE BETA SUBUNIT 1* (*PKpβ1*), *PDH E1 COMPONENT ALPHA SUBUNIT* (*PDH-E1*α), *BIOTIN CARBOXYL CARRIER PROTEIN 2* (*BCCP2*), *KAS1* and *ACYL CARRIER PROTEIN 5* (*ACP5*). Except for *PKp*α, the expression levels of the other genes in *PrASIL1*-transformed leaves were significantly lower than those in *P19*-transformed leaves; the expression level of *NbBCCP2* was reduced the most by 70% (Figure 4). The expression levels of several other FA and TAG biosynthesis genes were also down-regulated in *PrASIL1*-transformed leaves compared with the control leaves; these include *KETOACYL-ACP REDUCTASE* (*KAR*), *LIPOAMIDE DEHYDROGENASE1* (*LPD1*), *ENOYL-ACYL CARRIER PROTEIN REDUCTASE* (*MOD1*), *NAD-DEPENDENT G3P DEHYDROGENASE* (*GPDH*), *FAD2*, *FAD3*, *GPAT9*, and *DGAT1* but not *PDAT2* (Figure 4). These results confirm that the expression of *PrASIL1* induced down-regulation of several genes involved in FA and TAG biosynthesis.

### PrASIL1 Represses Seed Oil Accumulation and Alters Fatty Acids Composition in Transgenic Arabidopsis

**FIGURE 5:**
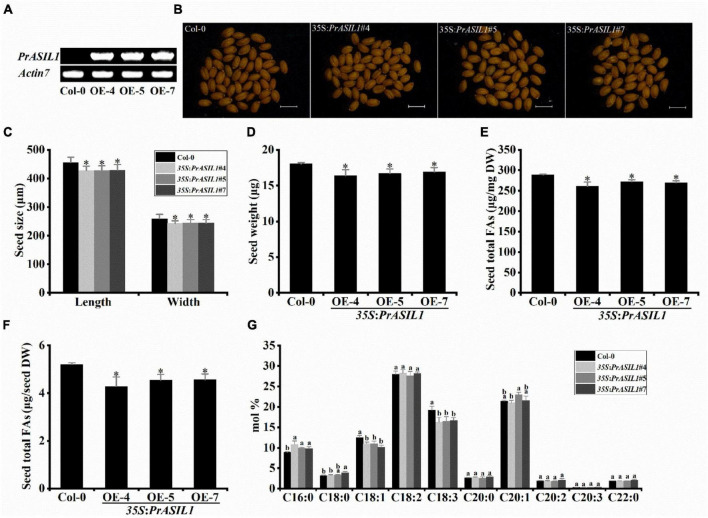
Overexpression of *PrASIL1* reduces seed oil content and alters FA composition in transgenic Arabidopsis. **(A)** RT-PCR analysis of *PrASIL1* expressed in transgenic Arabidopsis seed. **(B)** Microscopic observation of mature seeds randomly collected from wild-type (Col-0) and *PrASIL1* transgenic plants. Bars = 500μm. **(C)** Quantitative comparison of seed size (length and width) between the wild type (Col-0) and *PrASIL1* transgenic plants. **(D)** Quantitative comparison of the dry weight of seeds between the wild type (Col-0) and *PrASIL1* transgenic plants. **(E)** Comparison of total FA content (μg/mg) between the wild type (Col-0) and *PrASIL1* transgenic plants. **(F)** Comparison of total FA content (μg/dry seed) between the wild type (Col-0) and *PrASIL1* transgenic plants. **(G)** Fatty acid composition of wild type (Col-0) and *PrASIL1* transgenic plants. DW, Dry weight. Values are means ± SD (*n* = 3). Asterisks indicate significant differences in the seed size **(C)**, dry weight of seed **(D)**, seed total FA content **(E,F)** compared to that in the wild type (two-tailed paired Student’s *t*-test, *P* < 0.05). Different letters indicate significant difference in the contents of major seed FA compositions **(G)** compared to that in the wild type (one-way ANOVA with Tukey’s post-test, *P* < 0.05).

Since *PrASIL1* is mostly expressed in the seeds, we investigated the role of PrASIL1 in seed oil accumulation by stable expression of PrASIL1 in Arabidopsis. We transformed the *PrASIL1*-overexpression construct (*35S*:*PrASIL1*) into wild-type Arabidopsis (Columbia-0). The independent T3 homozygous transgenic lines were identified by RT-PCR, and three lines with relatively high expression of *PrASIL1* (OE-4, 5, and 7) were selected for further analysis (Figure 5A).

The overexpression of *PrASIL1* did not show any phenotypic variation during its development in any of the tissues even though it was expressed under a strong constitutive promoter CAMV35S, except in seeds. In the three OE lines, the size and weight of mature seed was reduced, compared to the wild-type Arabidopsis seeds (Figures 5B–D). The total and per seed FA content was also significantly less in OE lines than in wild-type (Figures 5E,F). Furthermore, the increase in total FA content was accompanied by an alteration in FA composition. Compared to the wild type, the composition of C18:1 and C18:3 was significantly lower in OE seeds, while the proportion of C16:0 was significantly elevated (Figure 5G). Interestingly, lack of any significant phenotypic differences in the OE seedlings or their development compared to the wild-type suggests a less effective involvement of ASIL1 in other physiological processes. Overall, these results suggest that the overexpression of PrASIL1 led to reduced seed oil accumulation and altered FA composition in mature seeds.

### PrASIL1 Represses the Expression of Fatty Acids and Oil Synthetic Genes Including Upstream Positive Regulators During Seed Maturation in Transgenic Arabidopsis

**FIGURE 6:**
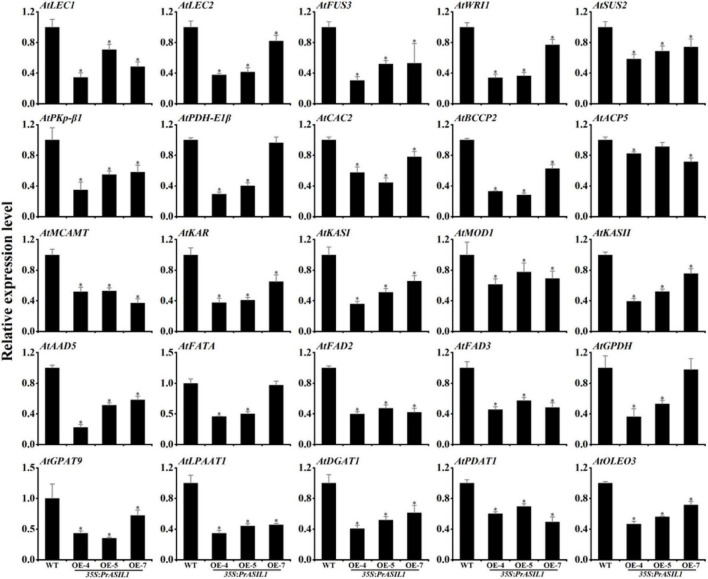
Comparison of the expression levels of genes involved in FA biosynthesis, modification, and TAG accumulation between the seed of wild type (Col-0) and *PrASIL1* transgenic plants. Results were normalized to the expression of *AtACTIN7* and calibrated to the levels of wild type. Values are mean ± SD (*n* = 3). Asterisks indicate significant differences in the expression levels compared to that in the wild type (two-tailed paired Student’s *t*-test, *P* < 0.05).

To identify the genes regulated by *PrASIL*1 in Arabidopsis, we examined the expression of various FA and TAG biosynthesis genes in seeds by qRT-PCR. First, we analyzed the expression levels of *WRI1*, *LEC1*, *LEC2*, and *FUS3*, the most important positive regulators involved in seed oil accumulation. The transcript levels of all these genes in transgenic seeds (12 DAP) were remarkably lower compared with wild-type, with subtle differences among the three transgenic lines (Figure 6). Second, the expression levels of 15 key FA biosynthetic genes were quantified. The transcript levels of *SUS2*, *PKp*-β*1*, *PDH-E1*β, *ACETYL CO-ENZYME A CARBOXYLASE BIOTIN CARBOXYLASE SUBUNIT 2* (*CAC2*), *BCCP2*, *MALONYL COA-ACP MALONYLTRANSFERASE* (*MCAMT*), *KAR*, *KASI*, *MOD*, *KASII*, *ACYL CARRIER PROTEIN DESATURASE 5* (*AAD5*), *FATTY ACYL-ACP THIOESTERASES A (FATA)*, *ACP5*, *FAD2*, and *FAD3* in transgenic seeds were significantly lower than those of wild type, except that *PDH-E1*β, *HAD*, *FATA*, and *ACP5* in OE-5 or OE-7 were not significantly lower than those of wild type (Figure 6). Third, six key TAG synthetic genes, including *GPDH*, *GPAT9*, *LPAAT1*, *DGAT1*, *PDAT1*, and *OLEOSIN* (*OLEO3*) were selected to analyze their transcript levels. Compared to wild-type seeds, their transcript levels in transgenic seeds were significantly lower, except the *GPDH* in OE-7 (Figure 6). Taken together, these findings also suggest that PrASIL1 inhibits the oil accumulation by repressing the expression of several FA and TAG synthetic genes including their upstream regulators, during seed maturation.

### Reduced Expression of *PrASIL1* in *Paeonia rockii* Enhances the Oil Content and Affects Fatty Acids Composition

**FIGURE 7:**
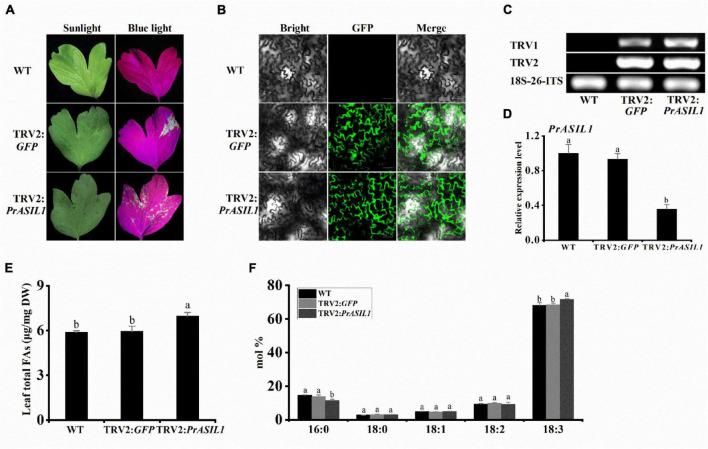
Reduced expression of *PrASIL1* in *Paeonia rockii* enhances the oil content and affects FA composition. **(A)** Image of *P. rockii* leaves infected with TRV2-*GFP* or TRV2-*PrASIL1* at 6 days post-infiltration under blue light. **(B)** Confocal microscopy image of *P. rockii* leaf tissue infected with TRV2-*GFP* or TRV2-*PrASIL1* at 6 days post-infiltration. Bars = 30μm. **(C)** RT-PCR analysis of *TRV1 and TRV2* accumulation levels in *P. rockii* leaf tissue at 2 weeks post-infiltration. **(D)** Silencing efficiency of *PrASIL1* determined by qRT-PCR analysis. Relative expression of *PrASIL1* was normalized to *18S-26S ITS* and calibrated to the levels of WT leaves. **(E)** Comparisons of total FA content (μg/mg) among the WT, TRV2:*GFP* control, and *PrASIL1*-silenced plants. **(F)** Fatty acid composition of WT, TRV2:*GFP* control, and *PrASIL1*-silenced plants. DW, Dry weight. Values are mean ± SD (*n* = 3). Different letters indicate significant difference at *P* < 0.05, as confirmed by one-way ANOVA with Tukey’s post-test.

To further validate the role of PrASIL1 in the regulation of oil accumulation in tree peony, we suppressed its endogenous expression in the leaves utilizing virus-induced gene silencing (VIGS) construct TRV2. GFP was used as a reporter and many green fluorescent spots were observed in the leaves inoculated with *TRV2*:*GFP* and *TRV2*:*PrASIL1* at 6 dpi, but not in the WT, under the excitation by blue light (Figure 7A) or using confocal microscopy (Figure 7B). The expression of *TRV1* and *TRV2* RNA was confirmed in the leaves of the inoculated line through RT-PCR (Figure 7C). Using qRT-PCR, we showed that the transcript level of endogenous *PrASIL1* in leaves inoculated with TRV2:*PrASIL1* was significantly lower than that of the WT and *TRV2*:*GFP* lines, and there was no significant difference between the latter two lines (Figure 7D).

Further, it is worth noting that the total FA content in *PrASIL1*-silenced leaves was much higher as the endogenous *PrASIL1* transcript levels decreased, relative to theTRV2:*GFP* control and the WT leaves (Figure 7E). Biochemical analyses revealed that the C16:0 composition was considerably lower, while the proportion of C18:3 was significantly increased, in the leaves of *PrASIL1*-silenced plants than those of the other two controls (Figure 7F). These results further confirmed that the expression level of *PrASIL1* can negatively affect the oil content and FA composition.

### *PrASIL1* Silencing in Tree Peony Increases the Transcript Abundance of Fatty Acids and Triacylglycerol Biosynthesis Genes

**FIGURE 8:**
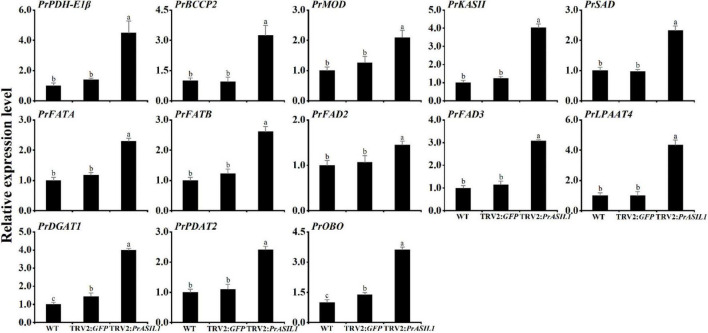
Comparison of the expression levels of FA and TAG biosynthetic genes among the WT, TRV2:*GFP* control, and *PrASIL1*-silenced plants. Relative expression of *PrASIL1* was normalized to *18S-26S ITS* and calibrated to the levels of WT leaves. Values are mean ± SD (*n* = 3). Different letters indicate significant difference at *P* < 0.05, as confirmed by one-way ANOVA with Tukey’s post-test.

From the above studies, it is clear that the overexpression of *PrASIL1* in *N. benthamiana* leaves and Arabidopsis seeds decreased the expression of FA and oil synthesis-related genes. Conversely, we studied the effects of silencing *PrASIL1* on genes contributing to FA and oil synthesis, which was opposite to its overexpression. The transcripts of FA biosynthesis genes, including *PDH-E1*α, *BCCP2*, *MOD1*, *KASII*, *SAD*, *FATA*, *FATB*, *FAD2*, and *FAD3*, were higher in the *PrASIL1*-silenced plants, relative to *TRV2*:*GFP* control and the WT lines (Figure 8). Similarly, genes involved in TAG assembly and accumulation, *LPAAT4*, *DGAT1*, *PDAT2*, and *OLEO* were also higher in *PrASIL1*-silenced plants compared to the controls; specifically, the expression level of *LPAAT4* was increased by three times and FAD3 expression was higher than FAD2 (Figure 8). All together, these results corroborate that the expression of *PrASIL1* is reciprocally associated with in FA and oil synthesis by affecting their gene expression.

## Discussion

The transcriptional regulation of the biosynthetic genes is a major factor affecting the supply of FA in TAG biosynthesis. Over the past decade, the transcriptional regulation of oil accumulation in many oil crops has been revealed. Overexpression of the endogenous *ZmLEC1* and *ZmWRI1* in maize markedly increased seed oil content (Shen et al., 2010). LEC2 from both castor and cocoa exhibits a function that enhance oil level (Kim et al., 2014; Zhang et al., 2014). Both *BnLEC1* and *BnWRI1* overexpression in *Brassica napus* significantly increased seed oil levels, While deletion of the *BnFUS3* resulted in reduced seed oil accumulation and affected the expression of *BnLEC1*, *BnLEC2*, *BnABI3*, and *BnWRI1* (Elahi et al., 2015, 2016; Li et al., 2015). Recently, a transcriptional regulatory network of soybean oil accumulation consisting of GmLEC1, GmLEC2, GmNFYA, GmZF392, GmZF351, GmLEC1, and GmWRI1 was identified, which involves multiple reactions of glycolysis, *de novo* FA synthesis, TAG assembly, and oil deposition to ultimately promotes oil accumulation (Lu et al., 2021). A regulatory network was also shown in oil palm, in which EgWRI1-1 is activated by three ABA-responsive transcription factors, EgNF-YA3, EgNF-YC2, and EgABI5, thereby promoting FA synthesis (Yeap et al., 2017).

In this present study, We found that a trihelix TF, *PrASIL1*, is highly expressed during early and late stage of seed maturation in tree peony (Figure 1C). This temporal expression coincides with the low rate of FA biosynthesis in the early and late stages of seed maturation (Zhang et al., 2018). Interestingly, the low *PrASIL1* expression in all the tissues examined, including those that do not accumulate much oil suggest that perhaps *PrASIL1* is expressed constitutively and associated primary FA metabolism and/or other functions. Previous studies have shown that trihelix TFs have multiple functions, such as in response to light and biotic and abiotic stresses (Gilmartin et al., 1992; Perisic and Lam, 1992; Osorio et al., 2012), plant morphogenesis, growth, and development (Tzafrir et al., 2004; Sun et al., 2015; Shibata et al., 2018). In Arabidopsis, AtASIL1 regulates a number of embryonic maturation genes and acts downstream of miRNAs to inhibit seed maturation (Gao et al., 2009; Willmann et al., 2011). It was inferred that ASIL1 might indirectly inhibit positive regulators of FA biosynthesis, such as LEC1, LEC2, FUS3, and ABI3 (Weselake et al., 2009). Phylogenetic analysis with Arabidopsis trihelix TFs showed that PrASIL1 is closest to AtASIL1, and there are four conserved motifs shared between them (Figure 2B), and are likely functionally conserved. But noteworthy, previous reports have shown that ASIL1 may repress the expression of LEC1, LEC2, FUS2, and ABI3 in Arabidopsis. In our study, overexpression of PrASIL1 was shown to reduce the expression of genes related to oil metabolism globally, including those derived from sucrose catabolism, *de novo* FA biosynthesis and modification, TAG assembly, and storage into the oil body as well as several master positive regulators.

Both transient and stable overexpression of *PrASIL1* induced a remarkable decrease of the total FAs and altered their compositions (Figures 3E,F, 5). Furthermore, stable expression of *PrASIL1* in Arabidopsis also resulted in a significant reduction in mature seed size, seed weight (Figure 5). Additionally, reverse genetics approach revealed that the silencing of *PrASIL1* leads to an increase in the levels of the total FAs and affected the composition of several major FAs (Figures 7E,F). In all the cases, the level of PUFA, especially C18:3, and FAD3 expression over FAD2 are highly affected. Thus, it is clear that a global shift in the expression of oil synthesis-related genes was induced by PrASIL1, which can impact both FA content and composition, specifically associated with PUFAs.

The coordinated expression of genes engaged in the oil biosynthesis is crucial for seed oil accumulation. The overexpression or silencing of *PrASIL1* affected the expression of several critical genes involved in multiple biological processes, such as glycolysis, FA synthesis and modification, and TAG assembly and accumulation, and thereby the oil accumulation. For example, SUS2 is a key enzyme for carbon metabolism in plant tissues, and directly participates in the sucrose catalytic reaction (Baroja-Fernandez et al., 2012). Similarly, ENO1 and PKp-β1 are functionally important in converting sucrose to acetyl-CoA, a precursor of the FA synthesis. Thus, the decreased expression of *SUS2*, *ENO1*, and *PKp*-β*1* in *PrASIL1*-overexpressed lines is likely to attenuate the production and distribution of photosynthetic products, thereby reducing the carbon source for oil accumulation.

Pyruvate produced by glycolytic metabolism in most oil-synthesizing tissues is converted into acetyl-CoA under the catalysis of PDHC, which is encoded by *PDH-E1*α, *PDH-E1*β, and *LPD1* (McGlew et al., 2015). Both *PDH-E1*α and *LPD1* were upregulated in *AtLEC1-* and *AtWRI1*-overexpressing plants, respectively, and were accompanied by an increase in lipid content (Mu et al., 2008; To et al., 2020). Similarly, the overexpression of *PrASIL1* decreased the expression of *NbPDH-E1*α and *NbLPD1* in tobacco and *AtPDH-E1*β in Arabidopsis, while the *PrPDH-E1*β was upregulated in *PrASIL1*-silenced tree peony. Next, the acetyl-CoA is catalyzed by ACCase to produce malonyl-CoA, a key step that determines the flow of FA biosynthesis in plastids (Mu et al., 2008). The transcripts for ACCase subunits, *CAC2* and *BCCP2* were significantly downregulated in *PrASIL11*-overexpressing transgenic Arabidopsis. Therefore, lower activity of ACCase in the initial stage of the FA biosynthetic pathway might inhibit subsequent oil accumulation in transgenic seeds. Thereafter, malonyl-CoA and ACP are catalyzed by fatty acid synthase (FAS) complex into C16:0-ACP, in which *ACP5*, *MCAMT*, *KASI*, *KAR*, *HAD*, and *MOD1* encode for the components (Li-Beisson et al., 2010). Our results showed that these genes involved in encoding FAS complex are down-regulated in *PrASIL1*- overexpressing transgenic Arabidopsis and tobacco, whereas the expression levels of *PrMOD1* is increased in *PrASIL1*-silenced tree peony. The considerable downregulation of these FAS-encoding genes should decelerate FA biosynthesis in *PrASIL11*-overexpressed plants. Finally, 16:0-ACP is elongated by KASII to 18:0-ACP and then desaturated by SAD to generate 18:1-ACP (Lindqvist et al., 1996; Carlsson et al., 2002). The content of C16:0 in Arabidopsis KASII mutant (*kas2*) was remarkably increased (Gao et al., 2020), whereas the decreased activity of AAD5 resulted in a significant reduction in the level of C18:1 in mature seeds (Kazaz et al., 2020). Thus, the decrease of *AtAAD5* expression led to the reduction of C18:1 in transgenic Arabidopsis, while the upregulation of *PrSAD* and *PrKASII* increased the C18:0 and C18:1 in *PrASIL1*-silenced tree peony. In the next step, Acyl-ACP thioesterases (FATs), such as FATA and FATB are required to catalyze the release of FA before they are transported from plastids to the endoplasmic reticulum for TAG synthesis. FATA generally releases ACP of the monounsaturated acyl chains with 18 carbons, while FATB acts on saturated acyl chains with 16 and 18 carbons (Jones et al., 1995; Dörmann et al., 2000; Salas and Ohlrogge, 2002). The reduction of FATA activity in Arabidopsis caused the decrease of oil content and change in FA composition of seeds (Moreno-Pérez et al., 2012). Consistently, the decline in C18:1 content of *PrASIL1*-overexpressed Arabidopsis seeds may be associated with the decrease in *AtFATA* expression, and the enhancement in C18:0 and C18:1 contents of *PrASIL1*-silenced plants may be due to the increased transcript levels of *PrFATA* and *PrFATB*. Hence, the suppression of several FA biosynthetic genes by PrASIL1 affects FA composition in transgenic plants.

The CoA esters synthesized in the plastid are transported to the ER that are modified by fatty acid desaturases. FAD2 is essential for polyunsaturated FAs biosynthesis from phospholipids (Okuley et al., 1994), while FAD3 possesses a crucial function in C18:3 biosynthesis (Shah et al., 1997). Therefore, the downregulation of endogenous *FAD2* and *FAD3* would individually inhibit the accumulation of C18:2 and C18:3 in transgenic Arabidopsis seeds and *PrASIL11*-overexpressed tobacco leaves. In contrast, increased levels of *PrFAD3* expression supported C18:3 accumulation in *PrASIL1*-silenced tree peony (Figure 7F). The effect of PrASIL1 was more pronounced on *FAD3* than *FAD2* either in overexpression or silenced conditions. Further, our results showed that PrASIL1 largely repressed the expression of TAG biosynthesis genes and TAG storage gene, such as endogenous *GPAT9*, *LPAAT1*, *DGAT1*, *PDAT2*, and *OLE3* (Figure 8). This could explain why over expression of *PrASIL1* in Arabidopsis seeds and *N. benthamiana* leaves accumulated significantly less oil than wild-type plants. Therefore, PrASIL1 has an inhibitory function on TAG biosynthesis and storage-related genes, which ultimately decreases the oil accumulation.

Among the network of major TFs, *ASIL1* was hypothesized as a negative regulator that indirectly down-regulates *WRI1*, *FUS3*, *LEC1*, and *LEC2*, which positively regulate seed maturation and oil accumulation in Arabidopsis (Weselake et al., 2009). Furthermore, LEC1 and LEC2 positively regulate *FUS3* (Kroj et al., 2003; Kagaya et al., 2005), and all three of them are upstream positive regulators of *WRI1* (Baud et al., 2007; Wang and Perry, 2013; Pelletier et al., 2017). As a master regulator of FA synthesis, WRI positively modulates the enzymatic reaction of glycolysis and FA biosynthesis by directly facilitating the expression of those genes (Focks and Benning, 1998; Cernac and Benning, 2004; Maeo et al., 2009). In transgenic Arabidopsis, the down-regulation of *WRI1*, *FUS3*, *LEC1*, and *LEC2* were accompanied by low expression levels of various oil biosynthetic genes and significant reduction in seed oil content. Similarly, the oil quantity of *PrASIL11*-overexpressed tobacco leaves, together with the transcript levels of *WRI1* and several oil biosynthetic genes, decreased (Figures 3E, 4). Since tree peony has no effective genetic transformation system, we used VIGS to silence *PrASIL1* in leaf tissues to predict its role in seed oil accumulation of tree peony. In agreement with the effects of heterologous overexpression of *PrASIL1*, the silencing of *PrASIL11* in tree peony leaves resulted in the increase of the expression level of oil synthetic genes along with the oil content.

## Conclusion

The trihelix transcription factor PrASIL1 is highly expressed in the early and late seed maturation phases indicating its active regulatory role in suppressing rate of seed oil biosynthesis in those phases. Structurally, PrASIL1 shares conserved domains with its ortholog from Arabidopsis, which indicates that both of them might be acting as a negative regulator of seed oil biosynthesis and seed maturation. To this extent, our results from transient and stable transgene over-expression in tobacco and Arabidopsis showed that PrASIL1 reduced total fatty acids and altered fatty acid compositions, which in turn associated with the decrease in expression levels of several genes involved in oil metabolism. Especially the expression of master positive regulators, such as *AtLEC1, AtLEC2, AtWRI1, and AtFUS3*, were repressed in transgenic Arabidopsis. Hence PrASIL1 may act as a negative regulator of oil biosynthesis, functioning upstream of master regulatory genes. Additionally, silencing of *PrASIL1* in tree peony leaves increased total fatty acids and affected fatty acid compositions are accompanied by increased expression of numerous genes in the oil biosynthesis pathway, which is in agreement with its negative role in oil metabolism. Taken together, PrASIL1 is likely to inhibit the expression of select oil biosynthesis genes by directly interacting with them or via the down-regulation of several master positive regulators. The mechanistic aspects of these interactions and target genes for PrASIL1 need further investigation. Nevertheless, it is clear that PrASIL1 acts as a negative regulator of oil accumulation at the transcription level and can be targeted for oil enhancement in other crops through gene manipulation.

## Data Availability Statement

The datasets presented in this study can be found in online repositories. The names of the repository/repositories and accession number(s) can be found in the article/Supplementary Material.

## Author Contributions

WY and JH carried out the experiments. WY analyzed the data and wrote the manuscript. LN, QZ, and YaZ conceived and designed the experiments. AK, JB, and LX analyzed the data and revised the manuscript. YY, YuZ, and YX assisted with doing the experiments. All authors read and approved the final manuscript.

## Conflict of Interest

The authors declare that the research was conducted in the absence of any commercial or financial relationships that could be construed as a potential conflict of interest.

## Publisher’s Note

All claims expressed in this article are solely those of the authors and do not necessarily represent those of their affiliated organizations, or those of the publisher, the editors and the reviewers. Any product that may be evaluated in this article, or claim that may be made by its manufacturer, is not guaranteed or endorsed by the publisher.

## Footnotes

1
http://www.clustal.org/download/2.0.11/

2
https://www.arabidopsis.org/

3
http://meme-suite.org/
