# Corrigendum: A Tree Peony Trihelix Transcription Factor PrASIL1 Represses Seed Oil Accumulation

**DOI:** 10.3389/fpls.2022.847770

**Published:** 2022-02-08

**Authors:** Weizong Yang, Jiayuan Hu, Jyoti R. Behera, Aruna Kilaru, Yanping Yuan, Yuhui Zhai, Yanfeng Xu, Lihang Xie, Yanlong Zhang, Qingyu Zhang, Lixin Niu

**Affiliations:** ^1^College of Landscape Architecture and Arts, Northwest A&F University, Yangling, China; ^2^Oil Peony Engineering Technology Research Center of National Forestry Administration, Yangling, China; ^3^Department of Biological Sciences, East Tennessee State University, Johnson City, TN, United States; ^4^Academy of Medical Sciences, Zhengzhou University, Zhengzhou, China

**Keywords:** PrASIL1, transcription factor, seed oil, tree peony, fatty acid biosynthesis

There is an error in the Funding statement. The correct number for **“Natural Science Foundation of China”** is **“31972453, 31901357, and 31971690**.” And the correct number for **“Postdoctoral Fund of Shaanxi Province”** is **“K3380220028.”**

The authors apologize for this error and state that this does not change the scientific conclusions of the article in any way. The original article has been updated.

## Publisher's Note

All claims expressed in this article are solely those of the authors and do not necessarily represent those of their affiliated organizations, or those of the publisher, the editors and the reviewers. Any product that may be evaluated in this article, or claim that may be made by its manufacturer, is not guaranteed or endorsed by the publisher.

