# Supplementary material for: A Tree Peony Trihelix Transcription Factor PrASIL1 Represses Seed Oil Accumulation
Source: Front Plant Sci. 2021 Dec 10;12:796181. doi: 10.3389/fpls.2021.796181 (PMC8702530; doi:10.3389/fpls.2021.796181)
Supplement: Supplementary file 1 [file Data_Sheet_1.docx]

Supplementary Material

# Supplementary Figures





**Supplementary Figure 1.** *PrASIL1*s transcription levels in Developing Seeds. RPKM: reads per kilobase per million.


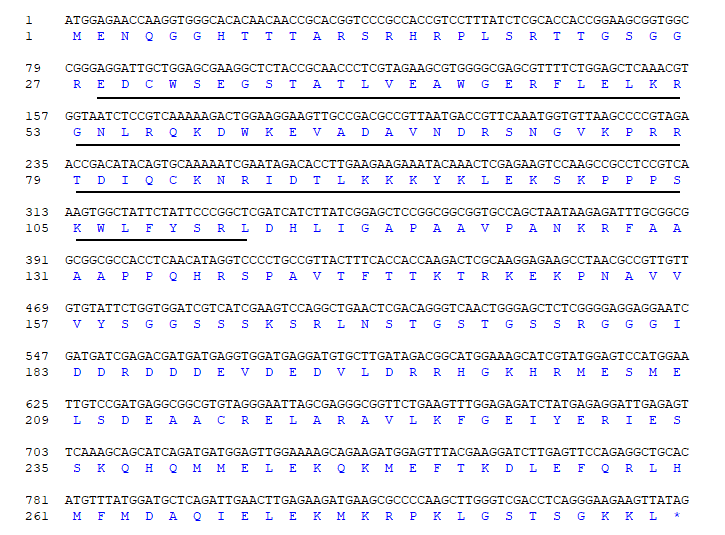


**Supplementary Figure 2.** Nucleotide and deduced amino acid sequence of PrASIL1. Solid lines indicate the Trihelix conserved domain.





**Supplementary Figure 3.** Motifs shared between the PrASIL1 and some Arabidopsis Trihelix TFs

# Supplementary Tables

**Supplementary Table 1.** Primers used for gene isolation and vector construction in the present study.

| **Primer name** | **Primer sequence (5’-3’)** |
| --- | --- |
| *PrASIL1-F* | TAAAACACTTCAGTCTATGGAG |
| *PrASIL1-R* | TTCAACAAACAAATCTCTCGTG |
| *35S:PrASIL1*-GFP-KpnI-F | GGggtaccCCATGGAGAACCAAGGTGGGCACAC |
| *35S:PrASIL1-GFP*--BamHI-R | CGggatccCGTAACTTCTTCCCTGAGGTCGAC |
| *2×35S:PrASIL1*-PstI-F | AActgcagAACCAAATGGAGAACCAAGGTGGGCACAC |
| *2×35S:PrASIL1*-BamHI-R | CGggatccCGCTATAACTTCTTCCCTGAGGTCG |
| *TRV2:PrASIL1*-BamHI-F | CGggatccCGCCTCCGTCAAAGTGGCTATTC |
| *TRV2:PrASIL1*-XhoI-R | CCGctcgagCGGCGTCTATCAAGCACATCCTCA |
| *35S:PrASIL1*-KpnI-F | GGggtaccCCATGGAGAACCAAGGTGGGCACAC |
| *35S:PrASIL1*--BamHI-R | CGggatccCGCTATAACTTCTTCCCTGAGGTCG |

**Supplementary Table 2.** Primers used for RT-PCR and qRT-PCR analysis in the present study.

| **Primer name and accession numbers** | **Primer sequence (5’-3’)** | **Annotation** |
| --- | --- | --- |
| *18S-26S ITS* | ACCGTTGATTCGCACAATTGGTCATCG | RT-PCR/qRT-PCR |
|  | TACTGCGGGTCGGCAATCGGACG |  |
| *PrASIL1* | CTGCCGTTACTTTCACCACC | qRT-PCR |
|  | GCACATCCTCATCCACCTCATC |  |
| *NbL23* (Niben101Scf01444g02009.1) | TGAGGACAACAATACCCTTG | RT-PCR/qRT-PCR |
|  | GTCCCATCAGGCCTAATCAA |  |
| *NbWRI1* (Niben101Scf04218g00013.1 ) | AGGCGTAGCAAGACACCACCATAA | qRT-PCR |
|  | ACCCACGATATTCTATAGCCGCCA |  |
| *NbSUS* (Niben101Scf02437g04010.1) | AATGCGCTCGTTGTTGAAGAGCTG | qRT-PCR |
|  | TCGCAGAAAGGTGCCTATTGAGGA |  |
| *NbENO1* (Niben101Scf11341g00010.1) | GCAGCACTAGACTCCAAAGC | qRT-PCR |
|  | GTGCTCCCGTCTTGATCTGT |  |
| *NbPKpα* (Niben101Scf08737g00001.1 ) | TAAAAGCTCGGGGCATGGTC | qRT-PCR |
|  | GGTTTCCCAGCTAAGAACTTGT |  |
| *NbPKpb1* (Niben101Scf06684g03025.1) | CTGTGTCGCTACGGACTGAA | qRT-PCR |
|  | GTGTTGGACATCATCGTTGC |  |
| *NbPDHE1α* (Niben101Scf06933g02041.1) | TGCACGAGATCCTATCAGTGCCTT | qRT-PCR |
|  | TACCTTCCATCAGGCCCAATTCCA |  |
| *NbBCCP2*(Niben101Scf12165g00003.1) | AACCACAGTTGCCTCCTGC | qRT-PCR |
|  | CCCTTTCTGGACCTTGTCTCC |  |
| *NbKASI* (Niben101Scf03455g05017.1) | ACCACATCGTTCACATCGAA | qRT-PCR |
|  | GAGTGGAGATGGACGGAAAG |  |
| *NbACP5*(Niben101Scf04146g02002.1 ) | TTGCTCCGTCTAGCTGTTCA | qRT-PCR |
|  | AATGCACCTGATTCTGCTTG |  |
| *NbKAR* (Niben101Scf05634g02005.1) | TCTCTCGCCTTTAATTCCGTCGCA | qRT-PCR |
|  | CAGCTTGTTCAGTTGTTGCCACCT |  |
| *NbLPD1* (Niben101Scf00466g04035.1) | TGCTGCTCTAATTGCGACTG | qRT-PCR |
|  | ACCAACTCTCCATTGGCATC |  |
| *NbMOD1* (Niben101Scf06661g04001.1) | GCCAAATGGTTCGTTGATG | qRT-PCR |
|  | TCCTGCATAGCGTTTGTTTG |  |
| *NbGPDH* (Niben101Scf01634g06003.1) | GGCGATCGGACACTATATGC | qRT-PCR |
|  | TTTCAGTCGAGGGCAATCC |  |
| *NbFAD2* (Niben101Scf06661g00011.1) | ACAATCCACCAGGCAGAATC | qRT-PCR |
|  | ATCAAAGTGGCAAGCGAACC |  |
| *NbFAD3* (Niben101Scf00318g10005.1) | GAGTTATCTTAGAGGAGGGC | qRT-PCR |
|  | CTTAGCTGCTTCAGTTGCTT |  |
| *NbGPAT9* (Niben101Scf04294g01001.1) | AGAACCTTGGAACTGGAACATTTAT | qRT-PCR |
|  | AGTTTTTTCCTGAACTTATCGTGCC |  |

**Continued Supplementary Table 2**

| *NbDGAT1* (Niben101Scf07042g00007.1) | CAATGTGCGTGCTTCTGTAT | qRT-PCR |
| --- | --- | --- |
|  | TCCCTTCCATCGGACTAACT |  |
| *NbPDAT2*(Niben101Scf07729g02007.1) | GCTTCTTATGACTGGAGGCTAT | qRT-PCR |
|  | TCCCATTGAATGAGGCACTA |  |
| *AtActin7(At5g09810)* | GGAACTGGAATGGTGAAGGCTG | RT-PCR/qRT-PCR |
|  | CGATTGGATACTTCAGAGTGAGGA |  |
| *AtLEC1*(AT1G21970) | GGCGCCGGTGACAAGA | qRT-PCR |
|  | GCCACACATGGTGGTTGCT |  |
| *AtLEC2*(AT1G28300) | TGGCAAGAGAGAGGTGGTTTTC | qRT-PCR |
|  | TCCTGTTGATCCTTGCCATCT |  |
| *AtFUS3*(AT3G26790) | GGTACTGGCCAAACAACAATAGC | qRT-PCR |
|  | CTAGCTGCAGACCATGAGCATT |  |
| *AtABI3*(AT3G24650) | GCAGGGATGGAAA | qRT-PCR |
|  | CACGTCGCTTTGCTTCAAGA |  |
| *AtWRI1(AT3G54320)* | CGCCGCCAGAGCAGTGGTTT | qRT-PCR |
|  | GCAGCAGCTTCCTCCTGCGT |  |
| *AtSUS2 (AT3G43190)* | TGCCATGAATAATGCCGATTTC | qRT-PCR |
|  | TTGCCCAACATTGTTCTTGCTT |  |
| *AtPKp-β1（AT5G52920）* | AGTCACTATCGTCCTTCCG | qRT-PCR |
|  | CTGTACGATTGCTATTTCCTC |  |
| *AtPDH E1β(AT1G30120）* | TGGAGCTGCCATGACTGGTCTA | qRT-PCR |
|  | TTTGGCGTTGTAAGGAGTTG |  |
| *AtCAC2(AT5G35360)* | TTCCGAATGTTCTGTCTGCGGCTA | qRT-PCR |
|  | TCGCTTTGTCTCCCATAACACGGA |  |
| *AtBCCP2(AT5G15530)* | AACCCAATGGGATCTCCTTTCCCT | qRT-PCR |
|  | ATAAATTCAGAGAGCTCGGCGGGT |  |
| *AtACP5(AT5G27200)* | TACGACGTTTGATGCCTGCT | qRT-PCR |
|  | GCTCTCTCTTCCGCCATTGT |  |
| *AtMCAMT(AT2G30200)* | GCTGATTACAAACCCACCTC | qRT-PCR |
|  | GCAAGTCACATCAACCGAGT |  |
| *AtKAR(AT1G24360)* | TGCCTCGAGAGGGATTGGTA | qRT-PCR |
|  | TCGCTTTCGAGACATCACCC |  |
| *AtKASI(AT5G46290)* | TCGATTTCAACTGCTTGTGC | qRT-PCR |
|  | CCTCCCAACCCAATAGGAAT |  |
| *AtMOD1(AT2G05990)* | TCAAGGAAAGGCTATCTCGCTGCT | qRT-PCR |
|  | TCACTCTCTAGTGCAGCTTTGGCA |  |
| *AtKASII*(AT1G74960) | TGCCTATCACATGACCGAGC | qRT-PCR |
|  | CCAAAACAGTGAGCAAGGGC |  |

**Continued Supplementary Table 2**

| *AtAAD5(AT3G02630)* | GGACCAGTTTTTAAGACCACGA | qRT-PCR |
| --- | --- | --- |
|  | ACATTATTGCCAAACCACTCTTT |  |
| *AtFATA(AT3G25110)* | AGCTGATCTCGACATGAACCAGCA | qRT-PCR |
|  | ATTTCAGAGGTGGTGGTGGTGAGT |  |
| *AtFAD2(AT3G12120)* | ATGGGTGCAGGTGGAAGAAT | qRT-PCR |
|  | CCAGGAGAAGTAAGGGACGA |  |
| *AtFAD3(AT2G29980)* | CCACAGTACTCGGATGCTCAGA | qRT-PCR |
|  | GCAATAAGCTTTCTCTCGCTTGGA |  |
| *AtGPDH(AT2G41540)* | GGGAGGTCTCAAGAATGTCTACGC | qRT-PCR |
|  | AGCAAAGGCCCTGCAAGTT |  |
| *AtGPAT9(AT5G60620)* | TCGGAAACCGGCGACGTAAGC | qRT-PCR |
|  | TGGCACCAGCAGCTTCAGTGAG |  |
| *AtLPAAT1(AT4G30580)* | GGTCGCATTTCTAATGGCATGACG | qRT-PCR |
|  | TTCACTCGGGACAGCATGAAGG |  |
| *AtDGAT1(AT2G19450)* | TCGCTCCCACATTGTGTTATCAGC | qRT-PCR |
|  | AAATTGACGAGCCACCCAACCC |  |
| *AtPDAT1(AT5G13640)* | AAAGGATGTTGCAGTTGCCAGAG | qRT-PCR |
|  | TGTTGAGTCCCATGTGCGTGTC |  |
| *AtOLEO3 (AT5G51210)* | CTCCTCGTCATCTTCAGCCC | qRT-PCR |
|  | ATCCAGATCCCGTCATGTGC |  |
| *TRV1* | CAGTCTATACACAGAAACAGA | RT-PCR |
|  | GACGTGTGTACTCAAGGGTT |  |
| *TRV2* | GGCTAACAGTGCTCTTGGTG | RT-PCR |
|  | GTATCGGACCTCCACTCGC |  |
| *PrPDH-E1β* | AGGCTGGCTTTACAGGGATT | qRT-PCR |
|  | GGGAATGTTGAGCACCAACT |  |
| *PrBCCP2* | GCCTCAATCTCTGTTCCATGCC | qRT-PCR |
|  | ATTTCAGCATAGCCCTCGCATT |  |
| *PrMOD* | ACTCAACCCTCAATGCCAAG | qRT-PCR |
|  | ATGTTGATTCCGCTCTCACC |  |
| *PrKAS II* | GAGATAGAGGCTTTTGATTGTG | qRT-PCR |
|  | ACCAGCAGTGAGCATATAAA |  |
| *PrSAD* | GCGTCATGAGACTGCCTACA | qRT-PCR |
|  | TCGCTGAGCTACAGACGAGA |  |
| *PrFATA* | CTTGGAAGTTTGACGGAGGA | qRT-PCR |
|  | AAGGTGCAATTTCCTCATGG |  |
| *PrFATB* | TCAGGTTCTTCGGGTAGGTT | qRT-PCR |
|  | CATCATCCATTGCTTCTCTGCC |  |

**Continued Supplementary Table 2**

| *PrFAD2* | TCACGCTTGGTGAAGTCAAG | qRT-PCR |
| --- | --- | --- |
|  | GTAGATGGGCCAAGCAACAT |  |
| *PrFAD3* | TCTTCCCTCAAATCCCACAC | qRT-PCR |
|  | GAGCTCATGGTCGGTCTTGT |  |
| *PrLPAAT* | ACAGCAGAAGCCAGTTGGAG | qRT-PCR |
|  | CGCAGGCATTATTTGTCCCG |  |
| *PrDGAT1* | TCCCGATTTTTCCTCTTGCCGC | qRT-PCR |
|  | CATACGAGGATAGGTTGGCTGG |  |
| *PrPDAT2* | AACGGTTGCTTGAAAGGTGGGG | qRT-PCR |
|  | CCCTATCACCTCCCATCTCTGC |  |
| *PrOLEO* | GGGATCCACAGTTGCTTGTT | qRT-PCR |
|  | GGCCGGAGAGGACTAAGAGT |  |
